# Assoication between self-reported sleep duration, physcial activity and the risk of all cause and cardiovascular diseases mortality from the NHANES database

**DOI:** 10.1186/s12872-023-03499-y

**Published:** 2023-09-18

**Authors:** Wenjie Wang, Jiaxin Yang, Kexin Wang, Jialong Niu, Jiang Wang, Zhi Luo, Hong Liu, Xiaoli Chen, Hailong Ge

**Affiliations:** 1 Department of Cardiology, Beijing Anzhen Hospital, Capital Medical University, Anzhen Avenue #2, Chaoyang District, Beijing, 100029 PR China; 2 Department of Cardiology, Beijing Geriatric Hospital, 100095 Beijing, PR China

**Keywords:** Sleep duration, Physical activity, Cardiovascular diseases, Risk of mortality

## Abstract

**Objective:**

The purpose of this study was to investigate the combined effect of self-reported sleep durations and physical activity (PA) on all cause and cardiovascular diseases mortality.

**Methods:**

Twenty-nine thousand fifty-eight participants (48.5% male, median age 49 years) from the National Health and Nutrition Examination Survey cycles 2007 to 2016 were included. We classified sleep duration into five categories (< 5.5 h/d,5.5–6.5 h/d,6.5–7.5 h/d,7.5–8.5 h/d, ≥ 8.5 h/d) and classified PA levels into three groups (high, medium and low). PA information and self-reported sleep duration were obtained by questionnaire. We derived 15 PA–sleep duration combinations. The primary endpoint was all-cause mortality, and the major secondary endpoint was cardiovascular diseases (CVD) mortality as of December 2022.

**Results:**

Median follow-up was 91 months. Compared with standard sleep duration (6.5–7.5 h/d), both shorter (< 5.5 h/d) and longer (≥ 8.5 h/d) sleep durations increased risks of all-cause mortality and CVD mortality in low PA. The deleterious associations of sleep duration with all outcomes was amplified by lower PA. There was no significant reduction in CVD mortality risk associated with increased physical activity during short sleep duration (< 6.5 h/d). During standard sleep, low PA significantly increased CVD mortality risk. At medium physical activity, both short and long sleep increased cardiovascular mortality. It was also found that sleep duration (≥ 8.5 h/d) was associated with a increase in all-cause and cardiovascular mortality at both low and high PA levels.

**Conclusions:**

This study suggested that low PA significantly increased the association of self-reported long and short sleep durations with all-cause and CVD mortality. All cause mortality appears to benefit from medium physical activity, while medium PA did not. Physical activity did not significantly reduce the risk of CVD mortality.

**Supplementary Information:**

The online version contains supplementary material available at 10.1186/s12872-023-03499-y.

## Introduction

Sleep and physical activity (PA) are an integral part of human life and are necessary to maintain our health. Sleep insufficient and physical inactivity may negatively impact the risk of all-cause mortality, cardiovascular disease (CVD) and cancer [1–3]. Sleep duration is influenced by many factors, such as diet, physical activity, genetics and environmental factors [4]. According to the results of the meta-analysis, mortality may be increased by long and short sleep durations compared with 7–8 h [5]. However, after sample analysis, there was no significant difference in mortality among patients with shorter sleep durations [5]. This suggests that longer sleep durations may be more likely to result in adverse outcomes compared to shorter sleep durations [6]. Compared with low PA, moderate and high PA reduced mortality [7]. Higher physical activity was strongly associated with lower CVD and mortality risks [7].

There is some evidence that sleep and PA are bidirectional. A meta-analysis found that moderate to vigorous PA was associated with better sleep quality in college students, possibly because moderate physical activity could treat insomnia [8]. However, sleep and PA are interdependent behaviors, with longer sleep times being associated with relatively less physical activity the next day. In contrast, another meta-analysis found no significant correlation between sleep and PA at the individual level in general [9]. According to our knowledge, there is little research on the relationship between PA and sleep duration with mortality [10]. Their potential joint effects are still largely unknown. Since both sleep duration and PA are associated with mortality, studying sleep duration and PA can better guide survival patterns. Therefore, we investigated the joint association of PA and sleep with mortality from the NHANES database.

## Methods

### Study design and participants

The datasets generated and analyzed in the current study are available on the NHANES website (https://www.cdc.gov/nchs/nhanes/index.htm). The National Health and Nutrition Examination Survey (NHANES) is a cross-sectional survey conducted by the National Center for Health Statistics of the Centers for Disease Control and Prevention to collect data on the health, nutrition, and health behaviors of the civilian noninstitutionalized population of the United States [11]. We downloaded five cycles of NHANES from 2007 to 2016 (2007–2008, 2009–2010, 2011–2012, 2013–2014, 2015–2016), demographic data and questionnaire data were obtained for all participants. To determine the survival status and cause of death of the enrolled participants, we used the NHANES public use linked mortality file as of December 31, 2018.

### Endpoints

The primary endpoint of this trial is all-cause mortality. The major secondary endpoint was CVD mortality. Additional endpoints were cerebrovascular diseases (CD) mortality, cardiovascular and cerebrovascular disease (CCD) mortality and non-CCD mortality. Deaths from any cause are included in the all-cause mortality. CVD (I00-I09, I11, I13, I20-I51) and CD (I60-I69) mortality are per the 10th Clinical Modification of the International Classification of Diseases [12].

### Physical activity and sleep

**Fig. 1:**
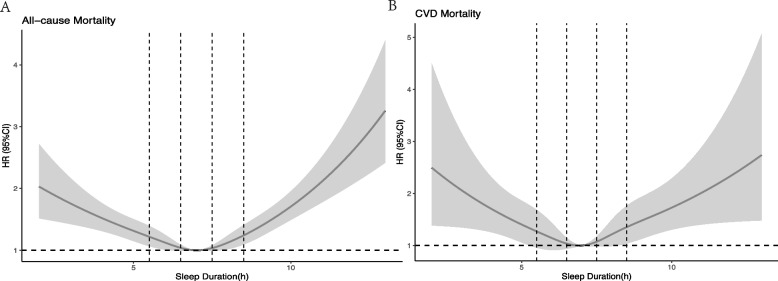
The restricted cubic spline curve between sleep duration and all-cause/CVD mortality

Information on PA was obtained by asking 5 questions about the different types of activities performed in a typical week. PA included having vigorous physical occupation/work, moderate physical occupation/work, walking/biking, vigorous recreation, and appropriate recreational activities. The metabolic equivalent (MET) score for a given activity was calculated based on the type and intensity of the activity [13, 14]. The Met score was multiplied by the average duration and number of times performed in the past 7 days to calculate Met minutes per activity per 7 days (Met min/7d). The MET min/7d for each activity was summed. PA is classified as low (0 to < 600 Met-min/week), medium (600 to < 1200 Met-min/week), and high (≥ 1200 Met-min/week) according to the WHO PA guidelines [15]. Nighttime self-reported sleep duration was obtained by answering the question "How much sleep do you usually get at night on workdays or weekdays? A restricted cubic spline (RCS) curve (Fig. 1) was used to model the nonlinear relationship between sleep duration and mortality, resulting in five categories of self-reported sleep duration (< 5.5 h/d, 5.5–6.5 h/d, 6.5–7.5 h/d, 7.5–8.5 h/d, ≥ 8.5 h/d) [16, 17].

### Covariate evaluation

The following demographic and lifestyle variables were obtained through a standardized questionnaire in the home interview: age, gender, education, marital status, smoking status, drinking status, work status, depression, and disease status (history of coronary heart disease, diabetes, hypertension). In addition, alcohol consumption was categorized by whether ≥ 4 drinks/day, smoking was categorized by whether ≥ 100 cigarettes in their lifetime, marriage was categorized by whether they were married or had a partner living together, and depression was categorized by whether Patient Health Questionnaire—9 ≥ 4.

### Statistical analysis

Normally distributed continuous variables were expressed as mean ± standard deviation, and non-normally distributed variables were presented as median and interquartile range (Q25, Q75). Categorical variables were expressed as numbers (percentages). Differences in continuous variables were analyzed by the t-test or Kruskal–Wallis test, and differences in categorical variables were assessed by χ2 test or Fisher's exact probability method. The relationship between sleep duration and mortality was then modeled using RCS curves, and sleep duration was finally categorized according to the results. The independent and joint associations of PA and sleep duration with mortality using cox proportional risk models. Our all data were weighted. We used Kaplan–Meier (KM) curve to examine the associations of physical activity and sleep duration with death from any all cause and cardiovascular death. We performed 2 models. Model 1 was an age- and gender-adjusted model; model 2 further adjusted for body mass index, education level, marital status, employment status, smoking status, drinking status, depression, sedentary behavior, baseline chronic diseases. Add sleep duration or PA to Model 2 as appropriate.

All statistical tests were two-sided, and *P* values < 0.05 were considered statistically significant. All statistical analyses were performed with R Software version 4.1.3 (R Foundation for Statistical Computing, Vienna, Austria) and IBM SPSS Statistics version 26.0 (IBM Corporation, Chicago, IL).

## Results

### Baseline characteristics of study participants

**Fig. 2:**
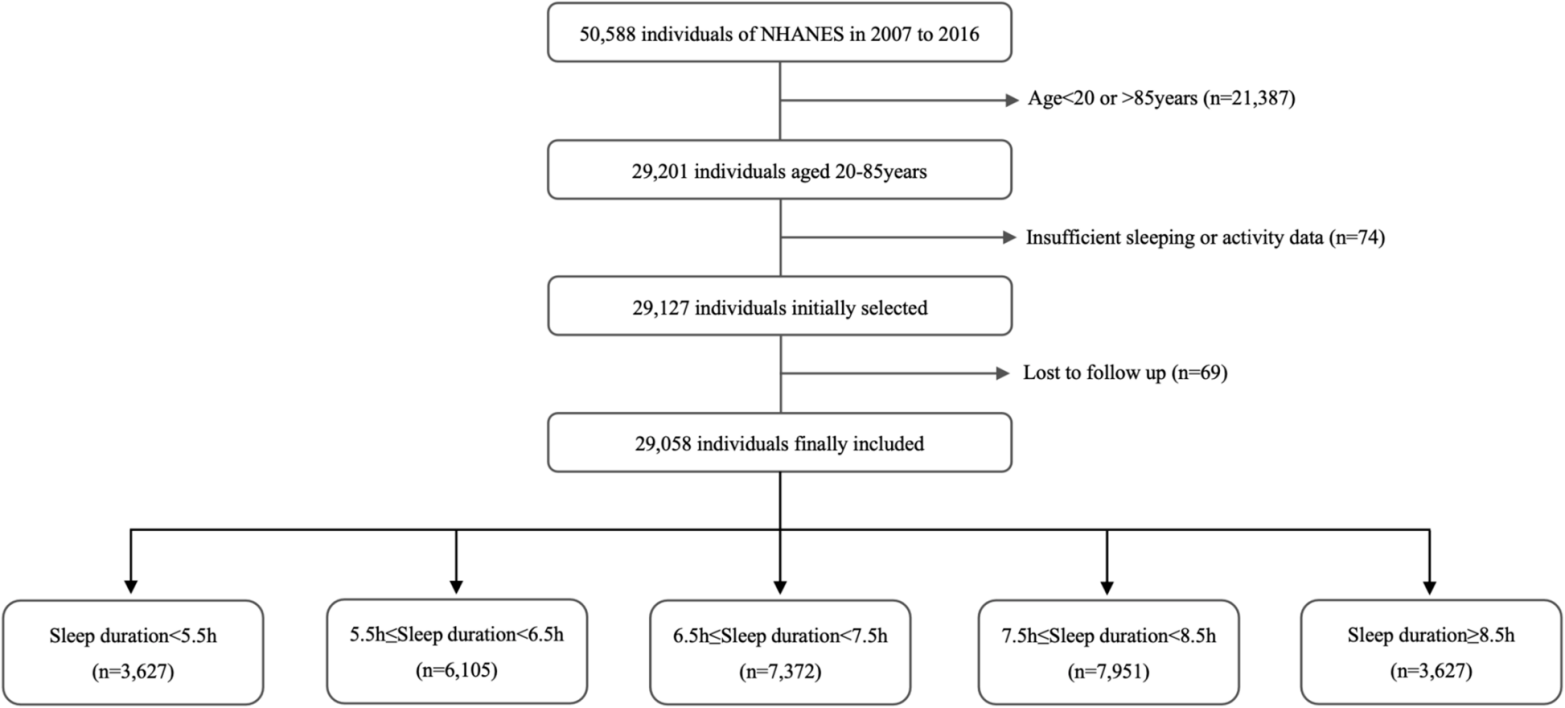
Flow chart of enrolment

Basic data on 50588 participants were derived from the NHANES database, 21,387 persons younger than 20 years old, 74 persons lacking sleep or physical activity, and 69 individuals lost to follow-up were excluded. A total of 29058 participants were included eventually and divided into 5 groups according to the different sleep duration (Fig. 2).

Among these participants, 48.5% were male, and the median age and BMI were 49 (34–64) years and 28.0 (24.28–32.5) kg/m2. The percentage of different PA levels are 63.4% (18410) low, 14.3% (4163) medium and 22.6% (6553) high respectively. In addition, a total of 3,337 (11.5%) participants died with CCD mortality accounting for 1001 (3.4%), including 822 (2.8%) caused by CVD and 179 (0.6%) caused by CD. And the remaining 2336 (8.0%) deaths were due to other causes. The largest number of individuals slept 7.5–8.5 h/d (27.4%), followed by 6.5–7.5 h/d (25.4%), 5.5–6.5/d (21%), < 5.5 h/d (13.8%) and ≥ 8.5 h/d (12.5%). There were significant differences in covariates for individuals with different sleep duration (*p* < 0.001) (Table 1).

**Table 1:** Baseline characteristics of the total population and stratified by groups of sleep duration

	All	Sleep duration (h/d)					*P* value
		< 5.5	5.5–6.5	6.5–7.5	7.5–8.5	≥ 8.5	
Number (n (%))	29058	4003 (13.8%)	6105 (21%)	7372 (25.4%)	7951 (27.4%)	3627 (12.5%)	
Age (years)	49 (34, 64)	49 (36, 62)	48 (35, 61)	48 (34, 62)	50 (34, 65)	54 (33, 71)	< 0.001
Male (n (%))	14096 (48.5)	1976 (49.4)	3063 (50.2)	3724 (50.5)	3767 (47.4)	1566 (43.2)	< 0.001
Body mass index (kg/m2)	28.0 (24.28, 32.5)	28.97 (24.8, 33.8)	28.21 (24.6, 32.9)	27.7 (24.07, 32.01)	27.7 (24.04, 31.99)	28.07 (24.18, 32.79)	< 0.001
Education level (n (%))							< 0.001
Less than 9th grade	3296 (11.3)	483 (12.1)	580 (9.5)	687 (9.3)	1005 (12.6)	541 (14.9)	
9-12th grade	19176 (65.9)	2975 (74.3)	4180 (68.5)	4554 (61.8)	5074 (63.8)	2393 (66.0)	
College or above	6586 (22.7)	545 (13.6)	1345 (22.0)	2131 (28.9)	1872 (23.5)	693 (19.1)	
Married or living with partner (n (%))	17053 (58.7)	2123 (53.0)	3514 (57.6)	4648 (63.0)	4812 (60.5)	1956 (53.9)	< 0.001
Employed in work (n (%))	15917 (54.8)	2088 (52.2)	3726 (61.0)	4680 (63.5)	4128 (51.9)	1295 (35.7)	< 0.001
Cigarette smoking^c^ (n (%))	12889 (44.4)	2079 (51.9)	2764 (45.3)	3072 (41.7)	3361 (42.3)	1613 (44.5)	< 0.001
Alcohol used^d^ (n (%))	17972 (61.8)	2400 (60.0)	3886 (63.7)	4726 (64.1)	4841 (60.9)	2119 (58.4)	0.001
Coronary artery disease^a^ (n (%))	2084 (7.2)	368 (9.2)	385 (6.3)	431 (5.8)	542 (6.8)	358 (9.9)	< 0.001
Hypertension (n (%))	10478 (36.1)	1726 (43.1)	2213 (36.2)	2348 (31.9)	2749 (34.6)	1442 (39.8)	< 0.001
Diabetes (n (%))	3730 (12.8)	614 (15.3)	778 (12.7)	767 (10.4)	975 (12.3)	596 (16.4)	< 0.001
Depression^b^ (n (%))	6304 (21.7)	1458 (36.4)	141 (2.3)	1185 (16.1)	1345 (16.9)	898 (24.8)	< 0.001
Sedentary behavior (min/d)	300 (180, 480)	300 (180, 480)	300 (180, 480)	300 (180, 480)	300 (180, 480)	360 (240, 480)	< 0.001
Physical active^e^ (n (%))							
Low	18410 (63.4)	2458 (61.4)	3703 (60.7)	4512 (61.2)	5140 (64.6)	2597 (71.6)	< 0.001
Medium	4163 (14.3)	483 (12.1)	886 (14.5)	1195 (16.2)	1184 (14.9)	415 (11.4)	< 0.001
High	6553 (22.6)	1069 (26.7)	1522 (24.9)	1686 (22.8)	1651 (20.8)	625 (17.2)	< 0.001
Outcomes							
All-cause mortality (n (%))	3337 (11.5)	536 (13.4)	613 (10.0)	625 (8.5)	977 (12.3)	586 (16.2)	< 0.001
CVD mortality (n (%))	822 (2.8)	130 (3.2)	146 (2.4)	138 (1.8)	246 (3.1)	162 (4.5)	< 0.001
CD mortality (n (%))	179 (0.6)	19 (0.4)	31 (0.5)	45 (0.6)	51 (0.6)	33 (0.9)	< 0.001
CCD mortality (n (%))	1001 (3.4)	149 (3.7)	177 (2.9)	183 (2.5)	297 (3.7)	195 (5.4)	< 0.001
Non-CCD mortality (n (%))	2336 (8.0)	387 (9.6)	436 (7.1)	442 (6.0)	680 (8.6)	391 (10.8)	< 0.001
Follow-up time (months)	89 (59,121)	97 (70,125)	98 (71,124)	94 (64,124)	86 (56,119)	56 (45,91)	< 0.001

*Abbreviations*: *CVD* cardiovascular disease, *CD* cerebrovascular disease, *CCD* cardiovascular and cerebrovascular disease

^a^Coronary heart disease, angina pectoris or heart attack

^b^Patient Health Questionnaire-9 > 4

^c^Smoked at least 100 cigarettes in life

^d^Drinked at least 12 alcohols per 1 year

^e^Categorization based on public health guidelines: low (< 600 MET-mins/week); medium (600–1200 MET-mins/week); and high (≥ 1200 MET-mins/week)

### Independent association of sleep duration and physical activity with mortality

**Fig. 3:**
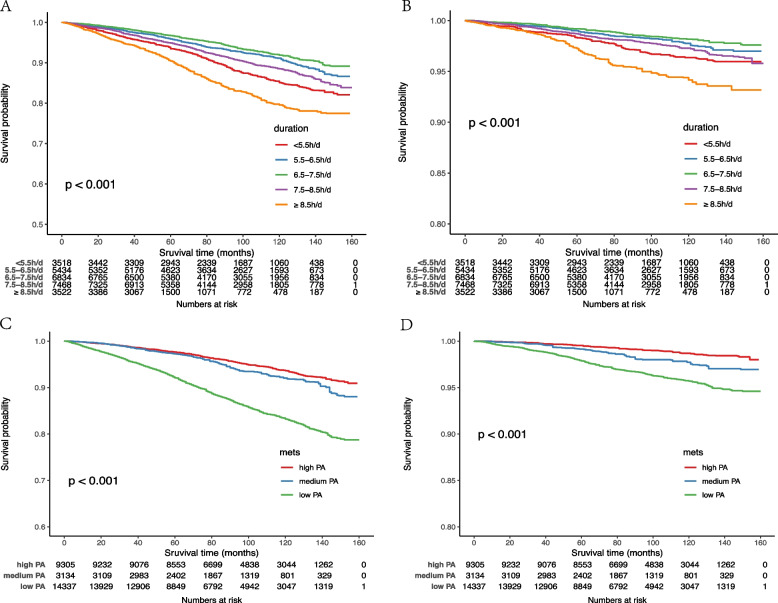
Kaplan–Meier curves of sleep duration and physical activity with all cause and CVD mortality

Figure 3 showed the Kaplan–Meier curves of sleep duration and physical activity with all cause and cardiovascular mortality. Supplement Table 3 showed the association of sex and sleep duration with all cause death mortality. We found that both increasing and decreasing sleep duration increased the risk of all-cause mortality and cardiovascular mortality, compared to sleep duration of 6.5–7.5 h/ day.

After adjusting for confounders (Table 2), compared with standard sleep duration (6.5–7.5 h/d), prolonged sleep duration (≥ 8.5 h/d) increased the risks of all-cause mortality [hazard ration (HR) = 1.436, 95%CI: 1.267–1.628], CVD mortality [HR = 1.556, 95%CI: 1.192–1.628], CCD mortality [HR = 1.454, 95%CI: 1.155–1.831], and non-CCD mortality [HR = 1.463, 95%CI:1.236–1.361]. At all endpoints, sleep duration < 5.5 h/d increased the risk of all-cause mortality [HR = 1.435, 95%CI: 1.243–1.657], CVD mortality [HR = 1.5, 95%CI: 1.10–2.050] and non-CCD mortality [HR = 1.463, 95%CI: 1.236–1.736] compared with standard sleep duration (6.5–7.5 h/d). However, the effect of different sleep durations on CD mortality was not statistically significant.

**Table 2:** Associations between sleep duration and mortality

Variable	Sleep duration, HR(95%CI)					*P* value for trend
	< 5.5 h/d	5.5–6.5 h/d	6.5–7.5 h/d	7.5–8.5 h/d	≥ 8.5 h/d	
All-cause mortality						
Model 1	2 (1.75,2.3)	1.28 (1.13,1.44)	1 [Reference]	1.33 (1.16,1.52)	1.84 (1.61,2.1)	< 0.001
Model 2	1.435 (1.243,1.657)	1.112 (0.996,1.265)	1 [Reference]	1.182 (1.039,1.345)	1.436 (1.267,1.628)	< 0.001
CVD mortality						
Model 1	2.14 (1.56,2.92)	1.37 (1.06,1.77)	1 [Reference]	1.33 (1.02,1.73)	1.95 (1.49,2.55)	0.003
Model 2	1.501 (1.10,2.050)	1.195 (0.919,1.554)	1 [Reference]	1.203 (0.924,1.565)	1.556 (1.192,2.03)	0.025
CD mortality						
Model 1	1.17 (0.66,2.06)	1.05 (0.55,2.02)	1 [Reference]	1.15 (0.78,1.7)	1.33 (0.71,2.51)	0.396
Model 2	0.841 (0.475,1.489)	0.919 (0.495,1.707)	1 [Reference]	1.029 (0.702,1.509)	1.078 (0.579,2.004)	0.597
CCD mortality						
Model 1	1.94 (1.49,2.52)	1.3 (1.05,1.61)	1 [Reference]	1.29 (1.05,1.6)	1.82 (1.45,2.29)	0.001
Model 2	1.365 (1.043,1.786)	1.138 (0.919,1.408)	1 [Reference]	1.166 (0.942,1.444)	1.454 (1.155,1.831)	0.021
Non-CCD mortality						
Model 1	2.03 (1.72,2.39)	1.27 (1.08,1.49)	1 [Reference]	1.34 (1.13,1.59)	1.83 (1.53,2.19)	< 0.001
Model 2	1.463 (1.236,1.736)	1.121 (0.954,1.317)	1 [Reference]	1.188 (1.004,1.405)	1.420 (1.197,1.683)	0.009

*Abbreviations*: *CVD* cardiovascular disease, *CD* cerebrovascular disease, *CCD* cardiovascular and cerebrovascular disease

Model 1. Minimally adjusted models were adjusted for age and sex

Model 2. Multivariable adjusted models were additionally adjusted for body mass index, education level, marital status, employment status, smoking status, drinking status, depression, sedentary behaviour, baseline chronic diseases and physical activity

Compared with high PA (Table 3), low PA had significant deleterious effects on all-cause mortality [adj.HR = 1.501, 95%CI: 1.322–1.705], CVD mortality [adj.HR = 1.588, 95%CI: 1.226–2.057], CD mortality [adj.HR = 1.762, 95%CI: 1.035–2.998], CCD mortality [adj.HR = 1.619, 95%CI: 1.275–2.055], and non-CCD mortality [adj.HR = 1.463, 95%CI:1.267—1.691], while all endpoints were not significantly affected by moderate physical activity.

**Table 3:** Associations Between Physical Activity and Mortality

Variable	Physical activity, HR(95%CI)			*P* value for trend
	≥ 1200METs	600-1200METs	< 600METs	
All-cause mortality				
Model 1	1 [Reference]	0.975 (0.805,1.18)	1.926 (1.698,2.186)	< 0.001
Model 2	1 [Reference]	0.892 (0.745,1.068)	1.501 (1.322,1.705)	< 0.001
CVD mortality				
Model 1	1 [Reference]	1.369 (0.991,1.891)	2.2 (1.710,2.839)	< 0.001
Model 2	1 [Reference]	1.195 (0.869,1.643)	1.588 (1.226,2.057)	0.001
CD mortality				
Model 1	1 [Reference]	1.181 (0.612,2.281)	2.237 (1.377,3.635)	0.001
Model 2	1 [Reference]	1.059 (0.54,2.078)	1.762 (1.035,2.998)	0.03
CCD mortality				
Model 1	1 [Reference]	1.337 (1.015,1.761)	2.211 (1.754,2.786)	< 0.001
Model 2	1 [Reference]	1.174 (0.891,1.547)	1.619 (1.275,2.055)	< 0.001
Non-CCD mortality				
Model 1	1 [Reference]	0.854 (0.675,1.08)	1.83 (1.596,2.099)	< 0.001
Model 2	1 [Reference]	0.794 (0.634,0.994)	1.464 (1.267,1.691)	< 0.001

*Abbreviations*: *CVD* cardiovascular disease, *CD* cerebrovascular disease, *CCD* cardiovascular and cerebrovascular disease

Model 1. Minimally adjusted models were adjusted for age and sex

Model 2. Multivariable adjusted models were additionally adjusted for body mass index, education level, marital status, employment status, smoking status, drinking status, depression, sedentary behaviour, baseline chronic diseases and sleep duration

### The joint relationship between sleep duration, physical activity, and mortality

**Fig. 4:**
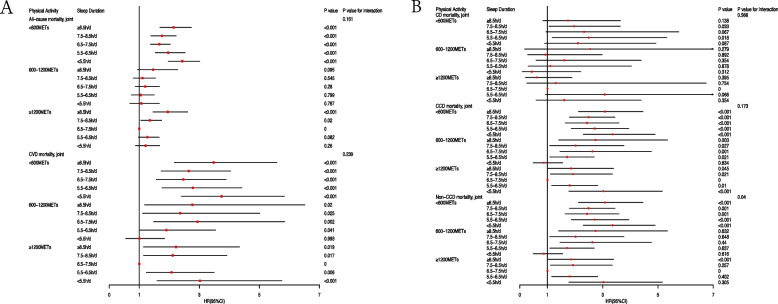
Joint association of physical activity and sleep duration with mortality

Figure 4 and Supplement Table 1 illustrated the HR for each exposure combination condition compared with the reference high PA sleep duration (6.5–7.5 h/d). There was no significant interaction between sleep duration and physical activity for all-cause mortality or CCD mortality [*P* value = 0.161, *P* value = 0.173]. However, there was a significant interaction between sleep duration and physical activity in non-CCD mortality (*P* value = 0.04). Any duration of sleep with low physical activity increased the risks of all-cause mortality, CVD mortality, CCD mortality, and non-CCD mortality.Sleep duration maintained a U-shaped association with all-cause mortality regardless of physical activity intensity, and those with low PA sleep duration < 5.5 h/d group had the highest CVD risks [adj.HR = 3.74, 95%CI: 2.39—5.84]. Compared with the high PA and sleep duration (6.5–7.5 h/d), moderate and low PA increased [12] the risk of CVD mortality. In all cause mortality, sleep duration ≥ 8.5 h/d, low PA and high PA were associated with mortality risk [HR = 2.15, 95%CI: 1.69–2.73, *P* value < 0.001; HR = 1.95, 95%CI: 1.46–2.61, *P* value < 0.001]. When sleep duration ≥ 8.5 h/d, high, moderate and low PA increased CVD mortality [HR = 3.48, 95%CI:2.16–5.58, *P* value < 0.001;HR = 2.77,95%CI:1.18–6.52, *P* value = 0.02, HR = 2.22, 95%CI: 1.14–4.34, *P* value = 0.019]. Sleep duration and physical activity did not significantly increase CD mortality, except when physical activity was low. Due to 80 percent of CCD mortality are CVD mortality, their results had similar trends. The risk of CVD mortality was not significantly reduced by increased physical activity during short sleep duration(< 6.5 h/d). When compared with high PA and a standard sleep duration, a low PA was associated with a significantly high mortality risk in all-cause mortality. In contrast to CVD mortality, moderate-to-vigorous PA reduced the risk of all-cause mortality at different sleep durations. Compared with standard sleep duration, increased or decreased sleep duration increased the risk of all-cause and CVD mortality in both low PA and high PA. On the other hand, no significant reduction in mortality risk was observed among moderate PA in CVD mortality.

## Discussion

In the large US cohort data, we examined the relationship between self-reported sleep duration and PA with mortality. The results suggest that both shorter (< 5.5 h/d) and longer (≥ 8.5/d) self-reported sleep durations increased risks of all-cause mortality and CVD mortality compared with standard sleep duration. Low PA had significant deleterious effects on all endpoints, and increased the association of long and short sleep durations with all-cause and CVD mortality. Shorter(< 6.5 h/d) sleep duration and increased physical activity did not significantly reduce CVD mortality risk. Moderate PA was associated with a reduced risk of death when sleep duration was longer, while vigorous PA did not. Increasing physical activity for shorter sleep duration did not significantly lower risk for cardiovascular mortality, but it did lower risk for all-cause mortality. Both low and high levels of PA were associated with increased  risk of all-cause and CVD mortality in different self-reported sleep duration.

There is a U-shaped correlation between sleep duration and the risk of death. A meta-study [18] concluded that the risk of death was lowest at 7 h of sleep, with a 6% increase in the risk of death for each hour of sleep increase and a 13% increase for each hour of sleep decrease [19]. Also, a study with a median follow-up of 25.9 years indicated that longer sleep duration was associated with significantly increased all-cause mortality. Yang [20] et al. found that both short (≤ 6 h/d) and long (≥ 8 h/d) sleep durations increased the risk of death compared to 7 h/d. However, a Korean study found that although prolonged sleep increased the risk of all-cause mortality, there was no significant relationship between prolonged sleep and CVD mortality and the exact mechanism was not clear. We divided sleep duration into 5 groups and indicated that the risks of all-cause mortality and CVD mortality were elevated for both longer and shorter sleep duration relative to 6.5–7.5 h/d, with the highest risk in sleep duration ≥ 8.5 h/d group. Both short and long sleep duration increased the risk of all-cause mortality, but after adjusting for self-rated health, the positive association with short sleep duration disappeared, and the positive association with long sleep duration weakened slightly. This suggests that the association of sleep mortality may be overestimated in studies that fail to control for subclinical disease. There was no significant positive association between long sleep duration and CVD mortality. Short sleep was found to be positively associated with specific CVD types (stroke, coronary heart disease), but not with total CVD mortality. It is possible that the association of sleep loss is specific to certain CVD types and that the number of disease-specific deaths among participants is limited [21]. Some studies have found that increasing physical activity can reduce the risk of CVD [22]. One study has found that increasing physical activity reduced mortality reduction in patients with and without CVD [23]. Another study showed [24] that high levels of moderate-to-vigorous physical activity reduced or even eliminated the risk of cardiovascular and cancer mortality associated with sedentary, especially in people who were sedentary in their daily activities.

Recent studies have shown a synergistic effect of physical inactivity and sleep deprivation on health [25–27]. One study [28] found Long sleep duration increased the risk of all-cause and CVD mortality, and in the 2 least physically active groups (< 15.0MET-h/week), all sleep duration groups were associated with elevated mortality risk, except the group that slept < 6 h/d and had 7.5–14.9 MET-h/week of PA. Another study found that sleep increases the risk of death, and that PA can further increase the risk of death, suggesting a synergistic effect between sleep and PA [2]. Our results generally agreed with it, suggesting that the deleterious associations of sleep duration with all outcomes was amplified by lower PA and participants with low PA and long sleep duration (≥ 8.5 h/d) were associated with the greatest risk of all-cause mortality. We divided people into 15 groups according to sleep duration and physical activity, and different groups had different risks of outcomes (for example, low physical activity and low sleep duration group), but there was no interaction between physical activity and sleep duration, and physical activity did not increase the effect of sleep on outcomes.

There have been several explanations for the relationship between sleep duration and health, with existing studies suggesting that sleep deprivation is associated with elevated levels of inflammatory biomarkers, chronic sleep deprivation (e.g. short sleep duration, sleep disorders) can lead to chronic low systemic inflammation and is associated with diseases of various inflammatory components, such as diabetes, atherosclerosis, and neurodegeneration, thereby increasing the risk of death [29–31]. A study indicated that [32] sleep duration and naps were associated with changes in lipid profiles and a larger waist circumference, which are common predisposing factors for stroke and cardiovascular disease [33]. Long sleep duration has also been associated with adverse cardiovascular diseases [34]. In addition, short sleep duration causes reciprocal changes in leptin and gastrin levels. These changes increase appetite and caloric intake, promoting the development of obesity and impaired glycemic control, thus contributing to the risk of cardiovascular disease [35]. And prolonged sleep restriction can lead to changes in cortisol secretion and growth hormone metabolic cycles. Excessive sleep duration causes immune dysfunction, fatigue and depression, systemic inflammation, the release of cytokines and alterations in several metabolic pathways, which raise the risk of heart disease [30, 35, 36]. PA may in turn reduce mortality from poor sleep patterns [37]. Within certain limits PA can promote sleep and improve sleep quality [38], but high PA can reduce sleep quality [39]. Another study found higher levels of coronary artery calcification and the probability of developing atrial fibrillation in very physically active individuals, which may cause higher mortality from cardiovascular disease [40]. Similar results were found in the present experiment, where the risks of all-cause and CCD mortality were not significantly lower in high PA with sleep duration ≥ 8.5 h/d compared to low PA with sleep duration ≥ 8.5 h/d, and even CVD mortality was elevated in high physical activity.

The strengths of this study include the large sample size and retrospective cohort design with long-term follow-up and extensive covariate measures, which allowed us to conduct several analyses to strengthen our interpretation. We adjusted for a wide range of sociodemographic and behavioral factors, and the present study allowed for a precise assessment of the relationship between sleep duration and PA by grouping sleep duration. Our study has some potential limitations. Our findings are based on data from the US population and this finding needs to be confirmed in other populations. Although obtaining sleep data of a large number of people through questionnaires has many advantages such as convenience, speed, and economy, it is indeed relatively subjective and not as objective and accurate as sleep monitoring or wearable device data. Also, the original data only included "sleep hours on weekdays" and there were no related questions about weekends, so this may have affected the results. Our study was a retrospective cohort study and therefore no causal inferences can be made.

## Conclusion

Sleeping too long or too short significantly increases risks of all-cause mortality and CVD mortality. The deleterious associations of sleep duration with all outcomes, except for CD mortality, was amplified by lower PA. The association between sleep duration and all-cause and CVD mortality was stronger in the low physical activity group than in the moderate physical activity group. The risk of CVD mortality was not significantly reduced by increasing physical activity when sleep duration was short. On the other hand, PA was found to reduce the risk of all-cause mortality. In both low and high PA groups, the risk of death decreased most with sleep duration of 6.5–7.5 h/d. Therefore, standard sleep duration combined with moderate-intensity exercise may be a better choice.

### Supplementary Information

**Additional file 1: Supplement Table 1.** Joint association of physical activity and sleep duration with mortality.** Supplement Table 2.** RCS table about sleep duration with CVD mortality. **Supplement Table 3.** Joint association of sex and sleep duration with mortality. **Supplement Figure 1.** The restricted cubic spline curve between sleep duration and other endpoints. 

## Data Availability

More information about the NHANES could be obtained at:http://www.cdc.gov/nhanes.

## Abbreviations

CD: Cerebrovascular diseases
CCD: Cardiovascular and cerebrovascular disease
CVD: Cardiovascular diseases
MET: Metabolic equivalent
NHANES: National Health and Nutrition Examination Survey
PA: Physical activity
RCS: Restricted cubic spline
