# Supplementary material for: Assoication between self-reported sleep duration, physcial activity and the risk of all cause and cardiovascular diseases mortality from the NHANES database
Source: BMC Cardiovasc Disord. 2023 Sep 18;23:467. doi: 10.1186/s12872-023-03499-y (PMC10508005; doi:10.1186/s12872-023-03499-y)

Supplement Table1. Joint association of physical activity and sleep duration with mortality.

| Physical Activity | Sleep Duration | HR(95%CI) | P value | P value for interaction |
| --- | --- | --- | --- | --- |
| **All-cause mortality, joint** |  |  |  | 0.161 |
| <600METs | ≥8.5h/d | 2.15(1.69,2.73) | <0.001 |  |
|  | 7.5-8.5h/d | 1.76(1.38,2.23) | <0.001 |  |
|  | 6.5-7.5h/d | 1.67(1.37,2.03) | <0.001 |  |
|  | 5.5-6.5h/d | 1.96(1.53,2.53) | <0.001 |  |
|  | <5.5h/d | 2.43(1.96,3.02) | <0.001 |  |
| 600-1200METs | ≥8.5h/d | 1.46(0.94,2.28) | 0.095 |  |
|  | 7.5-8.5h/d | 1.11(0.79,1.55) | 0.545 |  |
|  | 6.5-7.5h/d | 1.2(0.86,1.67) | 0.28 |  |
|  | 5.5-6.5h/d | 1.05(0.73,1.5) | 0.799 |  |
|  | <5.5h/d | 1.07(0.69,1.67) | 0.767 |  |
| ≥1200METs | ≥8.5h/d | 1.95(1.46,2.61) | <0.001 |  |
|  | 7.5-8.5h/d | 1.35(1.05,1.75) | 0.02 |  |
|  | 6.5-7.5h/d | Reference |  |  |
|  | 5.5-6.5h/d | 1.27(0.97,1.66) | 0.082 |  |
|  | <5.5h/d | 1.21(0.87,1.68) | 0.26 |  |
| **CVD mortality, joint** |  |  |  | 0.239 |
| <600METs | ≥8.5h/d | 3.48(2.16,5.58) | <0.001 |  |
|  | 7.5-8.5h/d | 2.64(1.73,4.05) | <0.001 |  |
|  | 6.5-7.5h/d | 2.47(1.56,3.9) | <0.001 |  |
|  | 5.5-6.5h/d | 2.79(1.76,4.42) | <0.001 |  |
|  | <5.5h/d | 3.74(2.39,5.84) | <0.001 |  |
| 600-1200METs | ≥8.5h/d | 2.77(1.18,6.52) | 0.02 |  |
|  | 7.5-8.5h/d | 2.36(1.11,5.02) | 0.025 |  |
|  | 6.5-7.5h/d | 2.94(1.48,5.85) | 0.002 |  |
|  | 5.5-6.5h/d | 1.9(1.02,3.54) | 0.041 |  |
|  | <5.5h/d | 1(0.55,1.84) | 0.993 |  |
| ≥1200METs | ≥8.5h/d | 2.22(1.14,4.34) | 0.019 |  |
|  | 7.5-8.5h/d | 2.11(1.14,3.92) | 0.017 |  |
|  | 6.5-7.5h/d | Reference | 0 |  |
|  | 5.5-6.5h/d | 2.08(1.23,3.5) | 0.006 |  |
|  | <5.5h/d | 3.02(1.59,5.76) | <0.001 |  |
| **CD mortality, joint** |  |  |  | 0.566 |
| <600METs | ≥8.5h/d | 1.74(0.84,3.64) | 0.138 |  |
|  | 7.5-8.5h/d | 1.96(1.06,3.64) | 0.033 |  |
|  | 6.5-7.5h/d | 2.33(0.94,5.75) | 0.067 |  |
|  | 5.5-6.5h/d | 2.5(1.17,5.32) | 0.018 |  |
|  | <5.5h/d | 2.1(0.9,4.93) | 0.087 |  |
| 600-1200METs | ≥8.5h/d | 2.55(0.47,13.86) | 0.279 |  |
|  | 7.5-8.5h/d | 0.92(0.29,2.98) | 0.892 |  |
|  | 6.5-7.5h/d | 1.61(0.59,4.39) | 0.354 |  |
|  | 5.5-6.5h/d | 1.11(0.3,4.04) | 0.878 |  |
|  | <5.5h/d | 0.43(0.08,2.21) | 0.312 |  |
| ≥1200METs | ≥8.5h/d | 0.61(0.2,1.89) | 0.395 |  |
|  | 7.5-8.5h/d | 1.3(0.25,6.74) | 0.754 |  |
|  | 6.5-7.5h/d | Reference | 0 |  |
|  | 5.5-6.5h/d | 3.07(0.93,10.19) | 0.066 |  |
|  | <5.5h/d | 1.61(0.59,4.39) | 0.354 |  |
| **CCD mortality, joint** |  |  |  | 0.173 |
| <600METs | ≥8.5h/d | 3.08(2.12,4.46) | <0.001 |  |
|  | 7.5-8.5h/d | 2.49(1.79,3.44) | <0.001 |  |
|  | 6.5-7.5h/d | 2.43(1.64,3.59) | <0.001 |  |
|  | 5.5-6.5h/d | 2.71(1.86,3.94) | <0.001 |  |
|  | <5.5h/d | 3.35(2.3,4.9) | <0.001 |  |
| 600-1200METs | ≥8.5h/d | 2.74(1.4,5.34) | 0.003 |  |
|  | 7.5-8.5h/d | 2.02(1.08,3.77) | 0.027 |  |
|  | 6.5-7.5h/d | 2.63(1.45,4.76) | 0.001 |  |
|  | 5.5-6.5h/d | 1.71(1.09,2.69) | 0.021 |  |
|  | <5.5h/d | 0.87(0.48,1.55) | 0.634 |  |
| ≥1200METs | ≥8.5h/d | 1.86(1.01,3.4) | 0.045 |  |
|  | 7.5-8.5h/d | 1.92(1.1,3.35) | 0.021 |  |
|  | 6.5-7.5h/d | Reference | 0 |  |
|  | 5.5-6.5h/d | 1.8(1.15,2.82) | 0.01 |  |
|  | <5.5h/d | 3.03(1.77,5.16) | <0.001 |  |
| **Non-CCD mortality, joint** |  |  |  | 0.04 |
| <600METs | ≥8.5h/d | 3.08(2.12,4.46) | <0.001 |  |
|  | 7.5-8.5h/d | 2.49(1.79,3.44) | 0.001 |  |
|  | 6.5-7.5h/d | 2.43(1.64,3.59) | 0.001 |  |
|  | 5.5-6.5h/d | 2.71(1.86,3.94) | <0.001 |  |
|  | <5.5h/d | 3.35(2.3,4.9) | <0.001 |  |
| 600-1200METs | ≥8.5h/d | 2.74(1.4,5.34) | 0.632 |  |
|  | 7.5-8.5h/d | 2.02(1.08,3.77) | 0.648 |  |
|  | 6.5-7.5h/d | 2.63(1.45,4.76) | 0.44 |  |
|  | 5.5-6.5h/d | 1.71(1.09,2.69) | 0.637 |  |
|  | <5.5h/d | 0.87(0.48,1.55) | 0.616 |  |
| ≥1200METs | ≥8.5h/d | 1.86(1.01,3.4) | <0.001 |  |
|  | 7.5-8.5h/d | 1.92(1.1,3.35) | 0.257 |  |
|  | 6.5-7.5h/d | Reference | 0 |  |
|  | 5.5-6.5h/d | 1.8(1.15,2.82) | 0.402 |  |
|  | <5.5h/d | 3.03(1.77,5.16) | 0.305 |  |

Supplement Table2. RCS table about sleep duration with CVD mortality.

|  | sleep | yhat | lower | upper |
| --- | --- | --- | --- | --- |
| 1 | 2 | 2.030547 | 1.5159857 | 2.719763 |
| 2 | 2.055276 | 2.014373 | 1.5110733 | 2.68531 |
| 3 | 2.110553 | 1.998328 | 1.5061438 | 2.651351 |
| 4 | 2.165829 | 1.982411 | 1.5011959 | 2.617882 |
| 5 | 2.221106 | 1.96662 | 1.496228 | 2.584898 |
| 6 | 2.276382 | 1.950956 | 1.4912387 | 2.552394 |
| 7 | 2.331658 | 1.935416 | 1.4862261 | 2.520366 |
| 8 | 2.386935 | 1.92 | 1.4811886 | 2.488811 |
| 9 | 2.442211 | 1.904706 | 1.4761243 | 2.457724 |
| 10 | 2.497487 | 1.889535 | 1.4710311 | 2.427101 |
| 11 | 2.552764 | 1.874484 | 1.4659069 | 2.396939 |
| 12 | 2.60804 | 1.859553 | 1.4607495 | 2.367235 |
| 13 | 2.663317 | 1.844741 | 1.4555564 | 2.337986 |
| 14 | 2.718593 | 1.830047 | 1.4503251 | 2.309188 |
| 15 | 2.773869 | 1.81547 | 1.4450529 | 2.280839 |
| 16 | 2.829146 | 1.80101 | 1.4397369 | 2.252936 |
| 17 | 2.884422 | 1.786664 | 1.4343739 | 2.225478 |
| 18 | 2.939698 | 1.772433 | 1.4289609 | 2.198463 |
| 19 | 2.994975 | 1.758315 | 1.4234942 | 2.171888 |
| 20 | 3.050251 | 1.744309 | 1.4179702 | 2.145753 |
| 21 | 3.105528 | 1.730415 | 1.412385 | 2.120057 |
| 22 | 3.160804 | 1.716632 | 1.4067345 | 2.094798 |
| 23 | 3.21608 | 1.702958 | 1.4010144 | 2.069977 |
| 24 | 3.271357 | 1.689394 | 1.3952199 | 2.045593 |
| 25 | 3.326633 | 1.675937 | 1.3893463 | 2.021646 |
| 26 | 3.38191 | 1.662588 | 1.3833883 | 1.998136 |
| 27 | 3.437186 | 1.649345 | 1.3773407 | 1.975066 |
| 28 | 3.492462 | 1.636207 | 1.3711977 | 1.952435 |
| 29 | 3.547739 | 1.623174 | 1.3649535 | 1.930245 |
| 30 | 3.603015 | 1.610245 | 1.358602 | 1.908499 |
| 31 | 3.658291 | 1.597419 | 1.3521367 | 1.887197 |
| 32 | 3.713568 | 1.584695 | 1.3455513 | 1.866342 |
| 33 | 3.768844 | 1.572073 | 1.338839 | 1.845937 |
| 34 | 3.824121 | 1.559551 | 1.3319929 | 1.825984 |
| 35 | 3.879397 | 1.547128 | 1.3250064 | 1.806487 |
| 36 | 3.934673 | 1.534805 | 1.3178725 | 1.787446 |
| 37 | 3.98995 | 1.52258 | 1.3105845 | 1.768867 |
| 38 | 4.045226 | 1.510452 | 1.3031359 | 1.75075 |
| 39 | 4.100503 | 1.498421 | 1.2955204 | 1.733099 |
| 40 | 4.155779 | 1.486485 | 1.2877321 | 1.715915 |
| 41 | 4.211055 | 1.474645 | 1.2797657 | 1.6992 |
| 42 | 4.266332 | 1.462899 | 1.2716166 | 1.682955 |
| 43 | 4.321608 | 1.451247 | 1.2632808 | 1.66718 |
| 44 | 4.376884 | 1.439687 | 1.2547554 | 1.651875 |
| 45 | 4.432161 | 1.428219 | 1.2460384 | 1.637037 |
| 46 | 4.487437 | 1.416843 | 1.2371289 | 1.622664 |
| 47 | 4.542714 | 1.405557 | 1.2280284 | 1.608751 |
| 48 | 4.59799 | 1.394358 | 1.2187466 | 1.595273 |
| 49 | 4.653266 | 1.383239 | 1.209301 | 1.582195 |
| 50 | 4.708543 | 1.372196 | 1.1997135 | 1.569476 |
| 51 | 4.763819 | 1.361224 | 1.19001 | 1.557071 |
| 52 | 4.819095 | 1.350318 | 1.1802192 | 1.544931 |
| 53 | 4.874372 | 1.339473 | 1.1703728 | 1.533005 |
| 54 | 4.929648 | 1.328684 | 1.1605041 | 1.521237 |
| 55 | 4.984925 | 1.317948 | 1.1506475 | 1.509574 |
| 56 | 5.040201 | 1.30726 | 1.140838 | 1.49796 |
| 57 | 5.095477 | 1.296616 | 1.1311108 | 1.486338 |
| 58 | 5.150754 | 1.286012 | 1.1215002 | 1.474655 |
| 59 | 5.20603 | 1.275443 | 1.1120401 | 1.462857 |
| 60 | 5.261307 | 1.264907 | 1.1027631 | 1.450892 |
| 61 | 5.316583 | 1.2544 | 1.0937007 | 1.438711 |
| 62 | 5.371859 | 1.243918 | 1.0848828 | 1.426266 |
| 63 | 5.427136 | 1.233457 | 1.0763381 | 1.413512 |
| 64 | 5.482412 | 1.223015 | 1.0680938 | 1.400408 |
| 65 | 5.537688 | 1.212589 | 1.0601755 | 1.386914 |
| 66 | 5.592965 | 1.202175 | 1.0526077 | 1.372996 |
| 67 | 5.648241 | 1.191772 | 1.0454136 | 1.35862 |
| 68 | 5.703518 | 1.181375 | 1.0386151 | 1.343757 |
| 69 | 5.758794 | 1.170983 | 1.0322331 | 1.328382 |
| 70 | 5.81407 | 1.160592 | 1.0262876 | 1.312473 |
| 71 | 5.869347 | 1.150202 | 1.0207975 | 1.296011 |
| 72 | 5.924623 | 1.139809 | 1.015781 | 1.278981 |
| 73 | 5.979899 | 1.129412 | 1.0112551 | 1.261374 |
| 74 | 6.035176 | 1.119011 | 1.0072345 | 1.243193 |
| 75 | 6.090452 | 1.108651 | 1.0037094 | 1.224566 |
| 76 | 6.145729 | 1.098403 | 1.0006538 | 1.2057 |
| 77 | 6.201005 | 1.088335 | 0.9980417 | 1.186797 |
| 78 | 6.256281 | 1.078514 | 0.9958478 | 1.168042 |
| 79 | 6.311558 | 1.069004 | 0.9940473 | 1.149613 |
| 80 | 6.366834 | 1.059868 | 0.992617 | 1.131675 |
| 81 | 6.422111 | 1.051164 | 0.9915347 | 1.11438 |
| 82 | 6.477387 | 1.042952 | 0.9907806 | 1.097871 |
| 83 | 6.532663 | 1.035287 | 0.9903373 | 1.082278 |
| 84 | 6.58794 | 1.028226 | 0.9901916 | 1.067721 |
| 85 | 6.643216 | 1.021822 | 0.9903356 | 1.054309 |
| 86 | 6.698492 | 1.016129 | 0.9907696 | 1.042138 |
| 87 | 6.753769 | 1.011202 | 0.9915062 | 1.03129 |
| 88 | 6.809045 | 1.007095 | 0.9925757 | 1.021828 |
| 89 | 6.864322 | 1.003863 | 0.9940354 | 1.013789 |
| 90 | 6.919598 | 1.001563 | 0.9959811 | 1.007176 |
| 91 | 6.974874 | 1.000252 | 0.9985615 | 1.001946 |
| 92 | 7.030151 | 0.999989 | 0.9979923 | 1.00199 |
| 93 | 7.085427 | 1.000774 | 0.9951015 | 1.006479 |
| 94 | 7.140704 | 1.002558 | 0.9930302 | 1.012176 |
| 95 | 7.19598 | 1.005291 | 0.9915932 | 1.019179 |
| 96 | 7.251256 | 1.008929 | 0.9906624 | 1.027532 |
| 97 | 7.306533 | 1.013425 | 0.9901569 | 1.037241 |
| 98 | 7.361809 | 1.018738 | 0.9900311 | 1.048277 |
| 99 | 7.417085 | 1.024824 | 0.9902647 | 1.060589 |
| 100 | 7.472362 | 1.031641 | 0.9908547 | 1.074107 |
| 101 | 7.527638 | 1.039149 | 0.9918099 | 1.088747 |
| 102 | 7.582915 | 1.047304 | 0.9931472 | 1.104415 |
| 103 | 7.638191 | 1.056066 | 0.9948891 | 1.121005 |
| 104 | 7.693467 | 1.06539 | 0.9970622 | 1.138401 |
| 105 | 7.748744 | 1.075233 | 0.9996958 | 1.156479 |
| 106 | 7.80402 | 1.08555 | 1.0028219 | 1.175102 |
| 107 | 7.859296 | 1.096293 | 1.0064741 | 1.194127 |
| 108 | 7.914573 | 1.107413 | 1.0106877 | 1.213395 |
| 109 | 7.969849 | 1.11886 | 1.0154993 | 1.232742 |
| 110 | 8.025126 | 1.130583 | 1.0209461 | 1.251993 |
| 111 | 8.080402 | 1.142551 | 1.0270428 | 1.271051 |
| 112 | 8.135678 | 1.154765 | 1.0337772 | 1.289913 |
| 113 | 8.190955 | 1.167224 | 1.0411349 | 1.308584 |
| 114 | 8.246231 | 1.17993 | 1.049101 | 1.327074 |
| 115 | 8.301508 | 1.192882 | 1.0576598 | 1.345393 |
| 116 | 8.356784 | 1.206081 | 1.0667949 | 1.363553 |
| 117 | 8.41206 | 1.219527 | 1.0764895 | 1.381569 |
| 118 | 8.467337 | 1.233219 | 1.0867258 | 1.39946 |
| 119 | 8.522613 | 1.247158 | 1.0974851 | 1.417244 |
| 120 | 8.577889 | 1.261345 | 1.1087479 | 1.434943 |
| 121 | 8.633166 | 1.275777 | 1.1204935 | 1.452581 |
| 122 | 8.688442 | 1.290456 | 1.1327001 | 1.470183 |
| 123 | 8.743719 | 1.30538 | 1.1453446 | 1.487777 |
| 124 | 8.798995 | 1.320549 | 1.1584028 | 1.505391 |
| 125 | 8.854271 | 1.335962 | 1.1718488 | 1.523058 |
| 126 | 8.909548 | 1.351618 | 1.1856557 | 1.54081 |
| 127 | 8.964824 | 1.367516 | 1.1997951 | 1.558682 |
| 128 | 9.020101 | 1.383654 | 1.2142375 | 1.576708 |
| 129 | 9.075377 | 1.400031 | 1.2289522 | 1.594926 |
| 130 | 9.130653 | 1.416646 | 1.2439077 | 1.613372 |
| 131 | 9.18593 | 1.433496 | 1.2590718 | 1.632085 |
| 132 | 9.241206 | 1.45058 | 1.2744119 | 1.651101 |
| 133 | 9.296482 | 1.467895 | 1.2898956 | 1.670457 |
| 134 | 9.351759 | 1.485438 | 1.3054907 | 1.690189 |
| 135 | 9.407035 | 1.503207 | 1.3211661 | 1.71033 |
| 136 | 9.462312 | 1.521199 | 1.3368916 | 1.730914 |
| 137 | 9.517588 | 1.53941 | 1.3526392 | 1.75197 |
| 138 | 9.572864 | 1.55784 | 1.3683908 | 1.773517 |
| 139 | 9.628141 | 1.57649 | 1.3841422 | 1.795567 |
| 140 | 9.683417 | 1.595363 | 1.3998913 | 1.81813 |
| 141 | 9.738693 | 1.614463 | 1.4156364 | 1.841215 |
| 142 | 9.79397 | 1.633791 | 1.4313762 | 1.86483 |
| 143 | 9.849246 | 1.653351 | 1.4471103 | 1.888984 |
| 144 | 9.904523 | 1.673145 | 1.4628386 | 1.913685 |
| 145 | 9.959799 | 1.693175 | 1.4785615 | 1.93894 |
| 146 | 10.015075 | 1.713446 | 1.4942798 | 1.964757 |
| 147 | 10.070352 | 1.733959 | 1.509995 | 1.991142 |
| 148 | 10.125628 | 1.754718 | 1.5257087 | 2.018101 |
| 149 | 10.180905 | 1.775725 | 1.541423 | 2.045642 |
| 150 | 10.236181 | 1.796984 | 1.5571403 | 2.07377 |
| 151 | 10.291457 | 1.818497 | 1.5728632 | 2.102492 |
| 152 | 10.346734 | 1.840268 | 1.5885946 | 2.131813 |
| 153 | 10.40201 | 1.8623 | 1.6043374 | 2.16174 |
| 154 | 10.457286 | 1.884595 | 1.620095 | 2.192278 |
| 155 | 10.512563 | 1.907157 | 1.6358705 | 2.223433 |
| 156 | 10.567839 | 1.92999 | 1.6516675 | 2.255212 |
| 157 | 10.623116 | 1.953095 | 1.6674893 | 2.287619 |
| 158 | 10.678392 | 1.976477 | 1.6833396 | 2.320663 |
| 159 | 10.733668 | 2.00014 | 1.6992217 | 2.354348 |
| 160 | 10.788945 | 2.024085 | 1.7151393 | 2.388681 |
| 161 | 10.844221 | 2.048317 | 1.7310958 | 2.423669 |
| 162 | 10.899497 | 2.07284 | 1.7470948 | 2.459319 |
| 163 | 10.954774 | 2.097655 | 1.7631395 | 2.495638 |
| 164 | 11.01005 | 2.122768 | 1.7792335 | 2.532633 |
| 165 | 11.065327 | 2.148182 | 1.79538 | 2.570311 |
| 166 | 11.120603 | 2.1739 | 1.8115824 | 2.608681 |
| 167 | 11.175879 | 2.199926 | 1.8278438 | 2.647749 |
| 168 | 11.231156 | 2.226263 | 1.8441672 | 2.687525 |
| 169 | 11.286432 | 2.252915 | 1.8605559 | 2.728017 |
| 170 | 11.341709 | 2.279887 | 1.8770128 | 2.769233 |
| 171 | 11.396985 | 2.307182 | 1.8935407 | 2.811182 |
| 172 | 11.452261 | 2.334803 | 1.9101426 | 2.853874 |
| 173 | 11.507538 | 2.362755 | 1.9268211 | 2.897317 |
| 174 | 11.562814 | 2.391042 | 1.9435791 | 2.941522 |
| 175 | 11.61809 | 2.419667 | 1.9604191 | 2.986499 |
| 176 | 11.673367 | 2.448635 | 1.9773436 | 3.032257 |
| 177 | 11.728643 | 2.47795 | 1.9943552 | 3.078808 |
| 178 | 11.78392 | 2.507616 | 2.0114564 | 3.126161 |
| 179 | 11.839196 | 2.537637 | 2.0286494 | 3.174329 |
| 180 | 11.894472 | 2.568017 | 2.0459367 | 3.223322 |
| 181 | 11.949749 | 2.598761 | 2.0633205 | 3.273151 |
| 182 | 12.005025 | 2.629873 | 2.0808029 | 3.323829 |
| 183 | 12.060302 | 2.661358 | 2.0983863 | 3.375368 |
| 184 | 12.115578 | 2.693219 | 2.1160727 | 3.42778 |
| 185 | 12.170854 | 2.725462 | 2.1338641 | 3.481077 |
| 186 | 12.226131 | 2.758091 | 2.1517627 | 3.535273 |
| 187 | 12.281407 | 2.791111 | 2.1697704 | 3.59038 |
| 188 | 12.336683 | 2.824526 | 2.1878892 | 3.646413 |
| 189 | 12.39196 | 2.858341 | 2.206121 | 3.703384 |
| 190 | 12.447236 | 2.892561 | 2.2244677 | 3.761308 |
| 191 | 12.502513 | 2.92719 | 2.2429312 | 3.820199 |
| 192 | 12.557789 | 2.962234 | 2.2615133 | 3.880071 |
| 193 | 12.613065 | 2.997698 | 2.2802158 | 3.94094 |
| 194 | 12.668342 | 3.033586 | 2.2990405 | 4.00282 |
| 195 | 12.723618 | 3.069904 | 2.3179892 | 4.065726 |
| 196 | 12.778894 | 3.106656 | 2.3370636 | 4.129676 |
| 197 | 12.834171 | 3.143849 | 2.3562654 | 4.194683 |
| 198 | 12.889447 | 3.181487 | 2.3755963 | 4.260766 |
| 199 | 12.944724 | 3.219575 | 2.395058 | 4.327939 |
| 200 | 13 | 3.25812 | 2.4146522 | 4.396221 |
|  |  |  |  |  |

Supplement Table3. Joint association of sex and sleep duration with mortality.

| Sex | Sleep Duration | HR(95%CI) | P value |
| --- | --- | --- | --- |
| **All-cause mortality** |  |  |  |
| Male | ≥8.5h/d | 3.16(2.55,3.92) | <0.001 |
|  | 7.5-8.5h/d | 1.7(1.44,2.02) | <0.001 |
|  | 6.5-7.5h/d | Reference |  |
|  | 5.5-6.5h/d | 1.25(1.06,1.49) | 0.009 |
|  | <5.5h/d | 2.14(1.8,2.55) | <0.001 |
| Female | ≥8.5h/d | 2.54(2.03,3.18) | <0.001 |
|  | 7.5-8.5h/d | 1.37(1.15,1.63) | <0.001 |
|  | 6.5-7.5h/d | Reference |  |
|  | 5.5-6.5h/d | 1.15(0.94,1.4) | 0.182 |
|  | <5.5h/d | 1.68(1.37,2.06) | <0.001 |
| **CVD mortality** |  |  |  |
| Male | ≥8.5h/d | 3.09(2,4.77) | <0.001 |
|  | 7.5-8.5h/d | 1.47(1.02,2.12) | 0.038 |
|  | 6.5-7.5h/d | Reference |  |
|  | 5.5-6.5h/d | 1.18(0.78,1.79) | 0.427 |
|  | <5.5h/d | 2.07(1.39,3.08) | <0.001 |
| Female | ≥8.5h/d | 3.73(2.53,5.51) | <0.001 |
|  | 7.5-8.5h/d | 1.8(1.23,2.63) | 0.002 |
|  | 6.5-7.5h/d | Reference |  |
|  | 5.5-6.5h/d | 1.39(0.99,1.95) | 0.055 |
|  | <5.5h/d | 1.99(1.3,3.06) | <0.001 |
| **CD mortality** |  |  |  |
| Male | ≥8.5h/d | 2.03(0.98,4.19) | 0.06 |
|  | 7.5-8.5h/d | 1.28(0.67,2.46) | 0.45 |
|  | 6.5-7.5h/d | Reference |  |
|  | 5.5-6.5h/d | 1.04(0.42,2.62) | 0.93 |
|  | <5.5h/d | 0.69(0.33,1.45) | 0.33 |
| Female | ≥8.5h/d | 2.85(1.18,6.87) | 0.02 |
|  | 7.5-8.5h/d | 1.59(0.82,3.07) | 0.17 |
|  | 6.5-7.5h/d | Reference |  |
|  | 5.5-6.5h/d | 0.87(0.36,2.09) | 0.76 |
|  | <5.5h/d | 1.56(0.58,4.19) | 0.37 |
| **CCD mortality** |  |  |  |
| Male | ≥8.5h/d | 2.89(2.02,4.12) | <0.001 |
|  | 7.5-8.5h/d | 1.43(1.05,1.95) | 0.022 |
|  | 6.5-7.5h/d | Reference |  |
|  | 5.5-6.5h/d | 1.16(0.84,1.6) | 0.381 |
|  | <5.5h/d | 1.8(1.27,2.54) | <0.001 |
| Female | ≥8.5h/d | 3.54(2.53,4.96) | <0.001 |
|  | 7.5-8.5h/d | 1.75(1.26,2.44) | <0.001 |
|  | 6.5-7.5h/d | Reference |  |
|  | 5.5-6.5h/d | 1.28(0.91,1.8) | 0.16 |
|  | <5.5h/d | 1.9(1.26,2.86) | 0.002 |
| **NCCD mortality** |  |  |  |
| Male | ≥8.5h/d | 3.29(2.5,4.27) | <0.001 |
|  | 7.5-8.5h/d | 1.83(1.45,2.31) | <0.001 |
|  | 6.5-7.5h/d | Reference |  |
|  | 5.5-6.5h/d | 1.3(1.01,1.67) | 0.038 |
|  | <5.5h/d | 2.31(1.84,2.89) | <0.001 |
| Female | ≥8.5h/d | 2.23(1.7,2.912) | <0.001 |
|  | 7.5-8.5h/d | 1.25(1.03,1.52) | 0.026 |
|  | 6.5-7.5h/d | Reference |  |
|  | 5.5-6.5h/d | 1.1(0.87,1.4) | 0.4069 |
|  | <5.5h/d | 1.61(1.29,2) | <0.001 |

Adjusted for age, gender, body mass index, education level, marital status, employment status, smoking status, drinking status, depression, sedentary behavior, baseline chronic diseases, and physical activity.

Supplement Figure1. The restricted cubic spline curve between sleep duration and other endpoints.


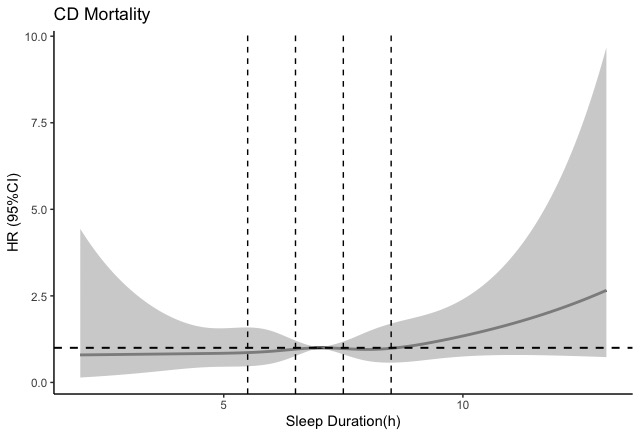

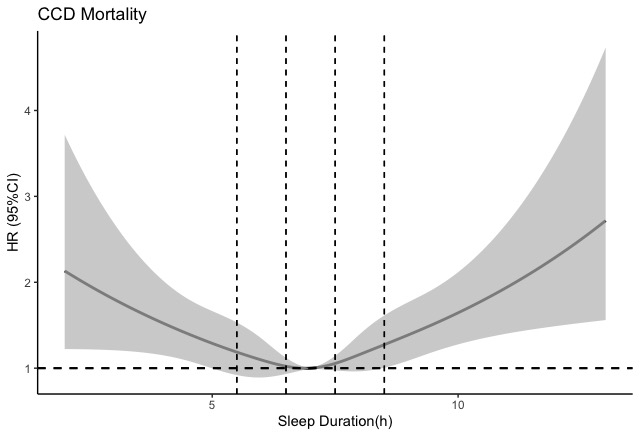


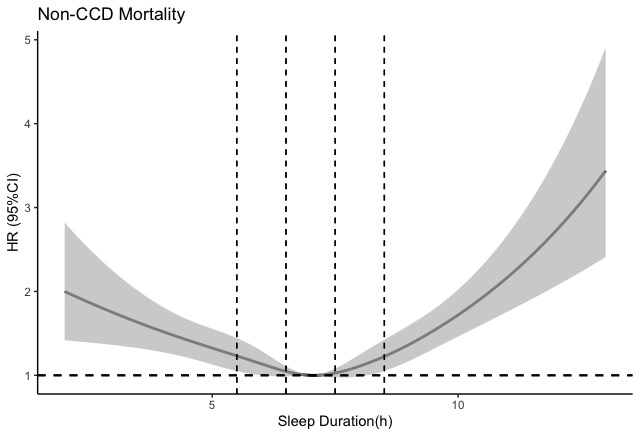

Supplement: Supplementary file 1 — Additional file 1: Supplement Table 1. Joint association of physical activity and sleep duration with mortality. Supplement Table 2. RCS table about sleep duration with CVD mortality. Supplement Table 3. Joint association of sex and sleep duration with mortality. Supplement Figure 1. The restricted cubic spline curve between sleep duration and other endpoints. [file 12872_2023_3499_MOESM1_ESM.docx]
